# Experimental Verification of Model-Based Wavefront Sensorless Adaptive Optics System for Large Aberrations

**DOI:** 10.3390/mi17010058

**Published:** 2025-12-31

**Authors:** Huizhen Yang, Yongqiang Miao, Peng Chen, Zhiguang Zhang, Zhaojun Yan

**Affiliations:** 1 School of Network & Telecom Engineering, Jinling Institute of Technology, Nanjing 211169, China; 2 School of Electronic Engineering, Jiangsu Ocean University, Lianyungang 222005, China; 3 The Astronomical Optical Instrument Group, Shanghai Astronomical Observatory, Chinese Academy of Sciences, Shanghai 200030, China

**Keywords:** adaptive optics, model-based method, deformable mirror, wavefront correction

## Abstract

To address the limitations of conventional wavefront sensorless adaptive optics (AO) systems regarding iteration efficiency and convergence speed, this study conducts an experimental validation of a model-based wavefront sensorless AO approach. A physical experimental platform was established, which consisted of a light source, a Shack–Hartmann wavefront sensor, a deformable mirror (DM), and an imaging detector. Wavefront aberrations under different turbulence levels were employed as correction objects to evaluate the performance of the model-based wavefront sensorless AO system. For comparative analysis, experimental results obtained by using the classical stochastic parallel gradient descent (SPGD) control algorithm are also presented. Under identical software and hardware conditions, the experimental results show that as the turbulence level increases, the SPGD-based wavefront sensorless AO system requires a larger number of iterations and exhibits a slower convergence. In contrast, the model-based wavefront sensorless AO system demonstrates improved applicability and robustness in correcting large aberrations under strong turbulence levels, maintaining an almost constant convergence speed and achieving better correction performance. These findings offer theoretical insights and technical support for the real-time correction potential of large wavefront aberrations.

## 1. Introduction

Compared with conventional adaptive optics (AO) methods [1], the wavefront sensorless AO system [2] does not require wavefront sensing, which significantly reduces the system complexity. Moreover, this method has broader applicability than traditional AO techniques and has been successfully applied in extended object imaging [3], medical imaging [4], beam shaping [5], and free-space optical communication [6]. Currently, this technology has become an active area of research in relevant fields.

According to the control algorithms employed, wavefront sensorless AO systems can be categorized into model-free and model-based approaches [7]. Model-free systems do not construct system models and directly utilize optimization algorithms, such as stochastic parallel gradient descent (SPGD) [8,9,10], the hill-climbing method [11], and simulated annealing [12], as controllers. These algorithms generally require hundreds of iterations, exhibit a slow convergence speed, and are sensitive to turbulence strength, which makes them difficult to be applied in near-real-time aberration correction systems [13]. Different from the model-free AO system, the model-based AO system first establishes a system model based on various principles, such as the modal method [14,15,16], non-linear optimization [7], and geometric optics [17], and then determines the corresponding system control method. Compared with the model-free control system, the model-based system has a faster convergence speed and greater potential for application in the field of near-real-time aberration correction.

The model-based method founded on geometric optics depends on a linear relationship between the second moment of the wavefront gradient and the masked far-field intensity to establish the system model. It is independent of specific basis functions and does not necessitate the prior removal of system aberrations, making the implementation relatively simple. Huang et al. put forward the proposition that there exists an approximate linear relationship between the masked intensity signal and the second moment of the wavefront gradient, and they indicated that this relationship is influenced by the detection radius [17]. Subsequently, Yang et al. rigorously deduced this linear relationship from physical optics, demonstrated that it can be represented with a fixed coefficient, and extended the formulation to extended object imaging [18]. Huang et al. applied the model-based approach to coherent beam combination experiments, thereby validating the feasibility of the algorithm [19]. Wen et al. proposed a synchronous model-based method through the improvement of the timing structure to reduce correction latency and experimentally verified it on a Fresnel Zone Plate imaging system [20]. Wang et al. experimentally verified the linear relationship between masked detector signals and the second moment of the wavefront gradient [21]. The majority of the existing experimental demonstrations of model-based wavefront sensor-less AO were conducted under relatively favorable laboratory conditions. These conditions typically involved low-order or small-amplitude aberrations, moderate turbulence strengths, and stable optical setups with limited noise. Such conditions do not sufficiently represent the performance of the model-based method under large aberrations and high levels of turbulence.

To address this issue, this paper establishes an experimental platform composed of a light source, a Shack–Hartmann wavefront sensor, a deformable mirror (DM), and an imaging detector. The linear relationship between the second moment of the wavefront gradient and the masked intensity distribution is incorporated into the feedback control loop to implement a model-based voltage update strategy, verifying the feasibility and efficiency of wavefront correction of the model-based AO system under different turbulence levels. For the convenience of comparison, the correction results of the classic control algorithm SPGD are also presented, offering a reference for the near-real-time correction application of large aberrations.

## 2. Model-Based Wavefront Sensorless AO Control Algorithm

The closed-loop control algorithm of the model-based AO system is constructed based on the linear relationship between the second moment of the wavefront gradient and the masked intensity distribution. This algorithm comprises a preprocessing stage and an iteration stage. In the preprocessing stage, the system response matrix and its inverse are computed for subsequent voltage determination. The mask can be generated by computer software, which is easy to implement. During the iteration process, the change in the performance metric is obtained through differential measurements, and the control signals is computed to update of the voltage and perform the closed-loop correction.

In the preprocessing stage, a set of basis functions that represent wavefront aberrations, such as Zernike polynomials or other orthogonal modes, is defined. For the purpose of illustration, Zernike polynomials are utilized as an example. Let ϕ(ε,η) represent the aberrant wavefront. The second moments of ε and η for each Zernike mode are computed and then inverted; this inverse matrix is denoted as *P* with its diagonal vector $P_{m}$. The influence functions of DM are experimentally measured, from which the coupling matrix that characterizes the interactions among influence functions is derived and denoted as $C_{v}$. This matrix $C_{v}$ is symmetric and invertible. Subsequently, the mutual relation matrix between DM influence functions and Zernike modes, denoted as $C_{zv}$, is calculated. The relationship between Zernike coefficients and the DM control voltage vector can be established [22] as follows:

(1) $$v^{*}={C_{v}}^{-1}C_{zv}Z,$$

where the DM control vector $v^{*}$ is the least-squares solution. It can be demonstrated that the optimal control voltages $v^{*}$ minimize the wavefront fitting error. Z denotes Zernike coefficient vector.

The primary steps of the iteration stage are as follows:

(1) Acquisition of far-field intensity. The imaging detector captures the corresponding far-field intensity distribution of the wavefront to be corrected. A region centered at the spot centroid is cropped, multiplied by the mask matrix M, and summed to yield the current masked intensity sum $I_{0}$. In the experiments, the mask is defined as
  (2) $$M=\left\{\begin{array}{cc} 1-(x^{2}+y^{2}), & \sqrt{x^{2}+y^{2}}\leq 1\\ 0, & others \end{array}\right.$$
  where (x,y) are the normalized spatial coordinates on the image plane.
(2) The voltages corresponding to each Zernike mode are applied to the DM to generate the perturbed wavefronts, and the corresponding masked intensities are recorded as $I_{1},I_{2},\ldots I_{N}$, *N* represents the number of Zernike modes, and ***a*** represent the vector of Zernike coefficients.
(3) Differential computation. The difference vector of the summed masked far-field intensities is denoted as ΔI:
  (3) $$\Delta I=[I_{1}-I_{0},\;I_{2}-I_{0},\;\ldots ,\;I_{N}-I_{0}]$$
(4) Compute the Zernike coefficients of the wavefront to be corrected using Equation (4), where $c_{0}$ is an adjustable parameter. Based on the linear relationship derived in reference [18], preliminary experiments were carried out to examine the influence of different $c_{0}$ values on convergence stability and correction accuracy. Ultimately, $c_{0}$ = 40 was selected as a fixed parameter for all experiments. At this value, the system converges stably without exhibiting oscillations in the later stages.
  (4) $$Z_{\left(1,\ldots ,N\right)}=\frac{P(c_{0}\Delta I-a^{2}P_{m})}{2*a}$$
(5) Obtain the control signal of the DM from Equation (1) and apply it to the DM.
(6) Iterative convergence. The residual wavefront after correction is regarded as the new input, and steps (1–6) are repeated until the predefined termination condition is fulfilled (e.g., maximum iteration count or intensity threshold).

It is worth noting that the vector $P_{m}$ and the matrices *P*, $C_{v}$, and $C_{zv}$ obtained in the preprocessing stage are independent of the wavefront aberration to be corrected. As a result, they can be computed in advance. Once the basis functions and the DM utilized in the system are fixed, these quantities can be directly reused as known parameters.

## 3. Experimental System

**Figure 1:**
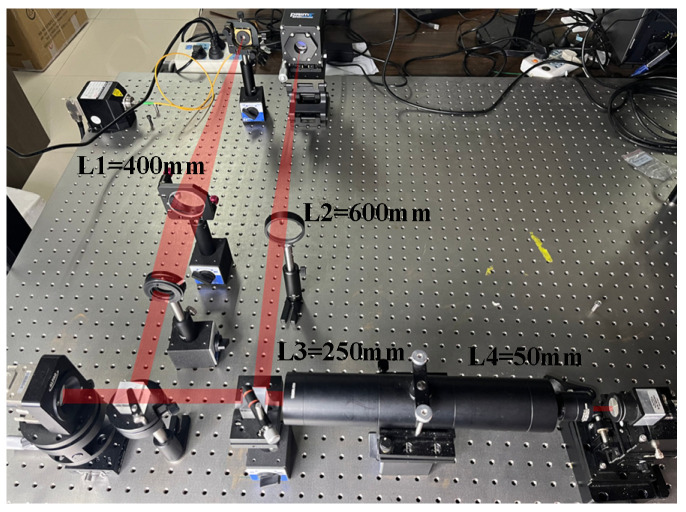
Model-based wavefront sensorless AO experimental setup.

**Figure 2:**
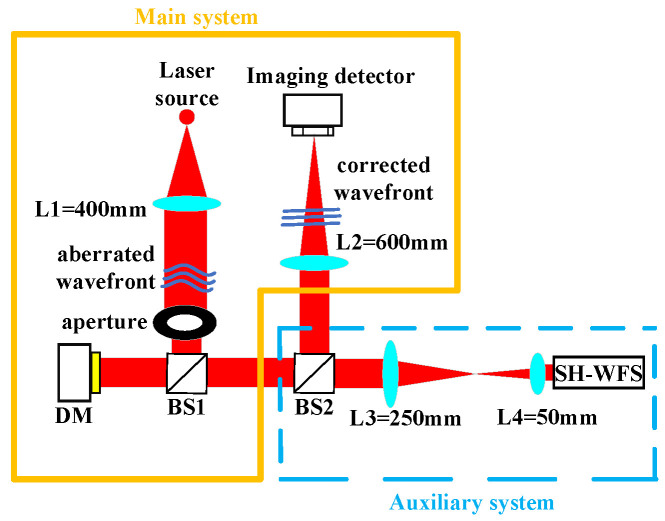
Schematic of the model-based wavefront sensorless AO system.

The experimental setup for the model-based wavefront sensorless AO system is depicted in Figure 1, while the corresponding optical schematic is presented in Figure 2. The primary components are as follows: an ALPAO 97-element (Bertin Alpao, Montbonnot, France) DM with a diameter of 25 mm and an effective aperture of 22.5 mm. A Shack–Hartmann wavefront sensor features 50 × 50 sub-apertures, a sub-aperture pitch of 96.6 µm, and a maximum frame rate of 28.14 kHz. The imaging detector is from Teledyne Photometrics and is equipped with a 2048 × 2048-pixel sensor having a pixel size of 6.5 µm × 6.5 µm. During experiments, the system operates at a 16-bit depth, leading to a frame rate of 43 frames per second (fps). The control software was developed using MATLAB R2022b, and all experiments and data processing were carried out on a workstation equipped with dual Intel(R) Xeon(R) Silver 4208 processors (2.10 GHz) and 64 GB of RAM.

After collimation by lens L1, the beam carrying wavefront aberrations is directed by beam splitter BS1 onto the DM and subsequently reflected. At beam splitter BS2, the beam is split into two separate optical paths. One path forms the closed-loop control path, where the beam is focused by lens L2 onto an imaging detector to capture the intensity distribution. The control computer acquires this intensity image and generates an updated control signal based on Equations (1)–(4). This signal is then amplified by a high-voltage driver and applied to the DM to compensate for the wavefront aberrations, thus achieving closed-loop correction. The other path functions as the wavefront measurement path. In this path, the beam propagates through a beam-reduction system composed of lenses L3 and L4 before entering the Hartmann wavefront sensor. It should be emphasized that the Hartmann wavefront sensor does not participate in the closed-loop control. Instead, the closed-loop control solely depends on the far-field intensity images provided by the imaging detector to achieve wavefront correction. The Hartmann wavefront sensor is mainly used to measure the influence function of the DM prior to the experiment to ensure the accuracy of the linear model relied upon by the model-based method.

To acquire the linear mapping between Zernike perturbations and the sums of masked intensity employed in the model control method, the influence functions of DM are initially measured using the Shack–Hartmann sensor. These measured functions are then utilized to construct the mapping matrix between Zernike coefficients and DM control voltages. Subsequently, the voltages corresponding to individual Zernike modes are calculated and sequentially applied to the DM. Meanwhile, the imaging detector records the resulting far-field intensity variations. Differential measurements of the perturbed intensities are used to estimate the system’s Zernike coefficients. Based on these coefficients, feedback voltages are derived and applied to the DM to carry out closed-loop wavefront compensation.

In the experiments, Mean Radius (MR) [23] and Peak Value (PV) are utilized as performance metrics, which are defined as follows:

(5) $$MR=\frac{\iint |(x,y)-(x^{\prime },y^{\prime })|I(x,y)dxdy}{\iint I(x,y)dxdy},$$

where $\left(x^{\prime },y^{\prime }\right)$ denotes the coordinates of the centroid of the image plane, calculated according to the following expression:

(6) $$x^{\prime }=\frac{\iint xI(x,y)dxdy}{\iint I(x,y)dxdy},y^{\prime }=\frac{\iint yI(x,y)dxdy}{\iint I(x,y)dxdy}.$$

Here $\left|\left(x,y\right)-\left(x^{\prime },y\prime \right)\right|$ represents the distance between each image pixel and the centroid and $I\left(x,y\right)$ is the far-field intensity distribution. A smaller MR value implies better correction performance. In the current experimental configuration, the source wavelength is λ = 635 nm, the effective aperture is D=22.5 mm, the detector pixel size is 6.5 µm, and the imaging focal length is f=600mm. The output power of the laser source is 2 mW. Based on the formula $d=\frac{2.44\lambda f}{D}$, the diffraction limit of this system is approximately 7 pixels.

To ensure a fair, time-based comparison of the convergence speeds of the model-based method and the SPGD method, this study employs the number of system iterations as a standardized metric. A system iteration is defined as one complete cycle where the DM undergoes a single deformation, the imaging detector conducts one sampling, and the control signal is calculated and then applied to the DM. When correcting N-order aberrations, each algorithmic iteration of the model-based method necessitates driving the deformable mirror N + 1 times and acquiring N + 1 far-field images, which corresponds to N + 1 system iterations. For the SPGD algorithm, the step size and perturbation amplitude were carefully selected based on preliminary experiments to guarantee stable convergence without oscillation. Specifically, the perturbation amplitude was set to 0.02 and the gain coefficient was 0.2. These parameters were kept constant for all experiments under different turbulence levels. The same termination criteria and hardware configuration were applied to both the model-based method and the SPGD algorithm to ensure a fair and consistent comparison. In contrast, each algorithmic iteration of SPGD involves two random perturbations (positive and negative), each of which requires one actuation of the DM, followed by an update of the control signal for the next actuation, resulting in three system iterations per algorithmic iteration. All performance evaluations presented in this paper utilize the number of system iterations as the abscissa to accurately represent actual time consumption and convergence efficiency.

## 4. Experimental Results and Analysis

Phase screens corresponding to different turbulence levels were generated using the Roddier method [24]. As tip/tilt are generally compensated separately by tip/tilt mirrors in AO systems, these components were excluded from the phase screens. Considering that the low-frequency aberrations account for the majority of wavefront aberrations caused by actual atmospheric turbulence, the 3rd to 20th order Zernike modes were selected as the research object. The generated phase screens statistically conform to the Kolmogorov spectrum and are mutually independent. Turbulence strength is characterized by the ratio $D/r_{0}$, where D denotes the aperture diameter of telescope and $r_{0}$ represents the atmospheric coherence length. A larger $D/r_{0}$ value corresponds to more severe wavefront aberration. Experiments were conducted under four different turbulent levels, namely $D/r_{0}=5,\;10,\;15,\;20$, corresponding to the wavefront aberrations increasing from small to large. At each turbulence level, 20 different phase screens were completed for closed-loop correction. During the closed-loop correction, the phase screen remains unchanged. However, owing to the imperfections of the experimental environment, slight disturbances occur during the light transmission process. It can be considered that the wavefront to be corrected is quasi-static aberration.

### 4.1. Aberration Correction Under Weak and Moderate Turbulence Levels ($D/r_{0}$ = 5 and 10)

**Figure 3:**
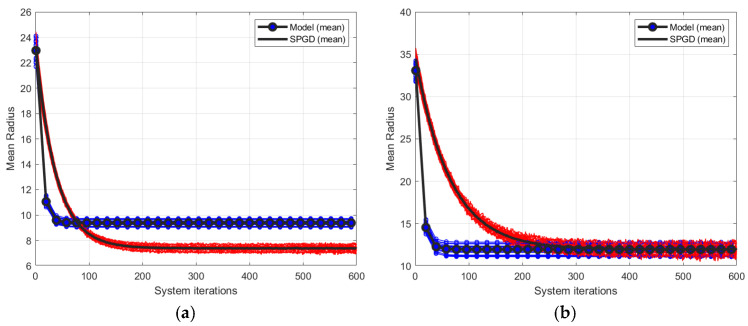
Closed-loop convergence curves of two control algorithms under weak and moderate turbulent level, where (**a**,**b**) correspond to $D/r_{0}=5$ and $D/r_{0}=10$ respectively.

Figure 3 illustrates the closed-loop convergence behavior of the model-based method and the SPGD algorithm under $D/r_{0}=5,\;10$, corresponding to weak and moderate turbulence levels. Figure 3a,b correspond to $D/r_{0}=5$ and 10, respectively, where the iteration curves represent the correction processes of 20 different phase screens for each turbulence level. The blue lines and red lines correspond to the model-based method and the SPGD algorithm, respectively, and the black solid lines denote the average of 20 different phase screens.

As depicted in Figure 3, under both turbulent conditions, both control algorithms achieved convergence after an adequate number of iterations. When turbulence is relatively weak, the convergence accuracy of the SPGD-based system is slightly higher than that of the model-based system. In the case of moderate turbulence level, the convergence values of the two control approaches are similar. Nevertheless, regardless of whether the turbulence is weak or moderate, the convergence speed of the model-based system is notably higher than that of the SPGD-based system.

**Figure 4:**
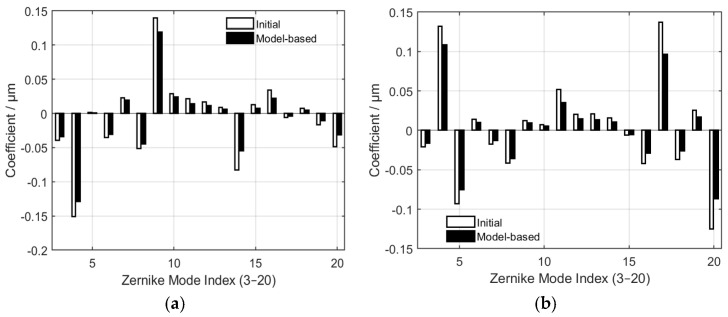
Comparison between the initial Zernike coefficients and those reconstructed by the model-based method for modes 3−20 under weak and moderate turbulent levels, where (**a**,**b**) correspond to $D/r_{0}=5$ and $D/r_{0}=10$ respectively.

To assess the correction accuracy of the model-based approach, a single frame was randomly chosen from 20 phase screens under each turbulence level. An analysis was conducted on the comparison of Zernike coefficients before and after correction by the control algorithm, as depicted in Figure 4, where (a) corresponds to $D/r_{0}=5$ and (b) is for $D/r_{0}=10$. The two datasets demonstrate consistent trends and signs, featuring amplitudes that are closely aligned with only insignificant deviations. This indicates that the model-based approach can accurately identify and reconstruct the dominant modal components of the wavefront aberration. Minor discrepancies are presumably attributable to inaccuracies in the measurement of the influence functions of DM or slight errors in the Shack–Hartmann sensor data. Overall, the minimal disparities confirm the validity and stability of the linear relationship assumed in the model, which is further supported by the reduction in MR values.

### 4.2. Aberration Correction Under Strong Turbulence Levels ($D/r_{0}=15$ and 20)

**Figure 5:**
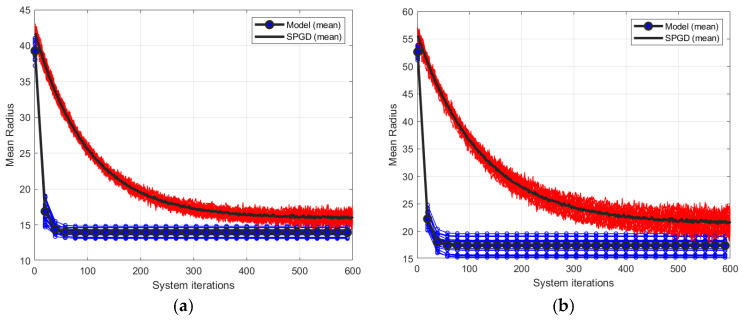
Closed-loop convergence curves of two control algorithms under strong turbulent levels, where (**a**,**b**) correspond to $D/r_{0}=15$ and $D/r_{0}=20$ respectively.

This subsection increases the turbulence level to investigate the correction and adaptability capabilities of the model-based AO system for large wavefront aberrations. The turbulence levels are $D/r_{0}=15$ and $D/r_{0}=20$ respectively. Figure 5 shows the closed-loop convergence behavior of the model-based method and the SPGD algorithm under two strong turbulence levels. Figure 5a,b correspond to $D/r_{0}=15$ and 20, respectively, where the iteration curves represent the correction processes of 20 different phase screens for each turbulence level. The blue lines and red lines correspond to the model-based method and the SPGD algorithm, respectively, and the black solid lines denote the average of 20 different phase screens.

As shown in Figure 5, under both turbulent conditions, both control algorithms reached convergence after a sufficient number of iterations. When compared with the curves in Figure 3, a notable difference is that under both strong turbulence levels, the convergence accuracy of the model-based system surpasses that of the SPGD-based system. Furthermore, the more severe the turbulence, the more pronounced this trend becomes (for comparison, refer to Figure 5a,b). Additionally, the model-based system exhibits a significantly faster convergence rate, with the value of MR decreasing rapidly within the first several tens of system iterations. In contrast, the SPGD-based system converges at a slower pace and requires substantially more iterations to reach a comparable correction performance. Evidently, the model-based method achieves superior performance in terms of both convergence speed and correction accuracy under strong turbulence levels.

**Figure 6:**
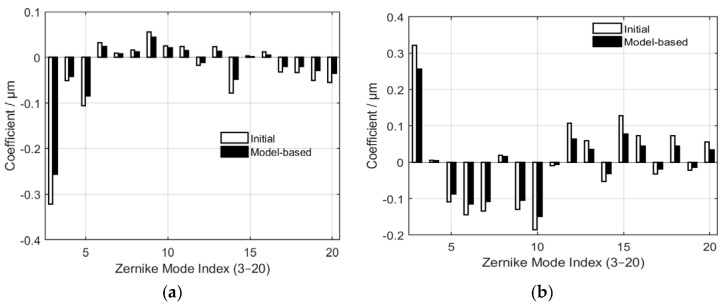
Comparison between the initial Zernike coefficients and those reconstructed by the model-based method for modes 3−20 under strong turbulent levels, where (**a**,**b**) correspond to $D/r_{0}=15$ and $D/r_{0}=20$ respectively.

Similarly, to assess the correction accuracy of the model-based approach, a single frame was randomly chosen from 20 phase screens under each turbulence level. An analysis was conducted on the comparison of Zernike coefficients before and after correction by the control algorithm, as depicted in Figure 6, where (a) and (b) correspond to $D/r_{0}=15$ and $D/r_{0}=20$ respectively.

As depicted in Figure 6, the two sets of data exhibit consistency in their overall distribution trends and sign patterns. This indicates that the model-based approach is capable of identifying and reconstructing the dominant modal components of the large wavefront aberrations. When compared with the weak or moderate turbulence levels (Figure 4), the discrepancies in the low-order modes become a little bigger, and the deviations in the high-order modes also display a slight increase. This phenomenon may stem from the substantial growth of the amplitudes of low-order aberrations under strong turbulence levels, which amplifies the impact of system noise and non-linear effects on the inversion results. Additionally, high-order modes are affected by factors such as the fitting accuracy of DM and optical path calibration errors, leading to increased residual errors. However, the differences between the two coefficient sets are still within an acceptable range, suggesting that the model-based method can maintain a reliable linear relationship under strong turbulence conditions. This result validates the feasibility of applying the method for wavefront correction in such environments.

### 4.3. Comparison of Correction Speeds Under Different Turbulence Levels

This section analyzes the convergence speed of the two algorithms based on two criteria: the number of system iterations required to reach 80% of their respective convergence values and the absolute elapsed time. The detailed comparison of results for both algorithms is presented as follows:

First, the time required for each system iteration is analyzed. Under the same software and hardware configurations, the average time per system iteration is approximately 0.67 s for the model-based system and approximately 0.69 s for the SPGD-based system. Thus, the two systems exhibit nearly equivalent computational efficiency per system iteration, indicating that their computational overheads in terms of hardware execution and signal-processing are comparable.

Secondly, we evaluate the number of system iterations required to achieve 80% of each method’s correction capability after 600 iterations. As shown in Figure 3a, under the weak turbulence level ($D/r_{0}=5$), the model-based method decreases MR from an initial value of 22 to 9, resulting in a total reduction of 13. This method reaches 80% of this reduction, which is equivalent to an MR of 11.6, within 19 system iterations. In contrast, the SPGD algorithm reduces MR from 22 to 7, a total reduction of 15; however, it requires 90 system iterations to reach 80% of this improvement, corresponding to an MR value of 12. Consequently, under weak turbulence level ($D/r_{0}=5$), the SPGD requires about 4.74 times more system iterations than the model-based method to achieve a comparable correction level. Similarly, SPGD requires about 8 times, 11.26 times and 14.53 times more system iterations than the model-based method when $D/r_{0}$ is 10, 15 and 20, respectively.

**Figure 7:**
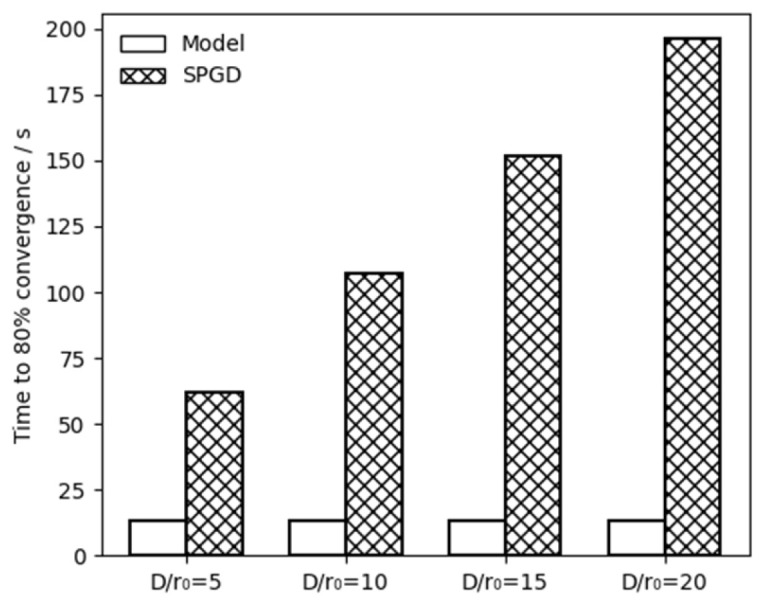
A comparison of the absolute time required for the convergence of the two systems under four different turbulence levels.

Finally, the absolute elapsed time of the two algorithms is compared. To clearly illustrate the time-efficiency advantage of the model-based method, Figure 7 presents the time required by the two algorithms to achieve 80% convergence under different turbulence levels. In this figure, blank bars represent the model-based method, while hatched bars denote SPGD algorithm. As shown in Figure 7, there are significant differences in the cumulative time between the model-based system and the SPGD-based system. As previously analyzed, the model-based method achieves 80% of MR reduction after 19 system iterations, with a total elapsed time of 13 s. In contrast, the numbers of system iteration needed by SPGD are 90, 152, 214 and 276 when $D/r_{0}$ is 10, 15 and 20, respectively. The corresponding total time are 62, 107, 152 and 196 s, respectively. The superior convergence speed of the model-based approach is primarily attributed to the significantly reduced number of system iterations. Moreover, this performance advantage becomes more pronounced as the turbulence level increases.

## 5. Discussion

This work experimentally validates a model-based wavefront sensorless adaptive optics control strategy, with a focus on convergence behavior, robustness, and applicability under large aberrations and different turbulence levels. In addition to the quantitative results, several practical aspects are discussed, such as the selection of a comparison baseline, computational characteristics, performance under strong turbulence levels, and potential for real-time implementation.

First, the computational requirements of the two algorithms are analyzed. During a system iteration process, the difference between the two control algorithms lies in the calculation of the control signal of DM. The model-based method consists of constructing the perturbation difference vector ΔI and computing the Zernike coefficients through a pre-calibrated linear mapping. The subsequent matrix–vector multiplication involves only a limited number of Zernike modes and therefore imposes minimal computational cost. The computational load of the SPGD method is mainly reflected in the calculation of the performance metric MR. In practice, the overall iteration time in both methods is dominated by hardware-related processes, including the response time of deformable mirror, the exposure time of imaging detector, and the time of data transfer. As analyzed in Section 4.3, under the same software and hardware configuration, the single-iteration time of the two algorithms differs by 0.02 s. However, since the number of iterations required for the model-based method to converge is far less than that required by SPGD, the difference in the time needed for a single system iteration can be disregarded.

Secondly, under strong turbulence levels, discrepancies are observed in the reconstruction of low-order Zernike modes. According to the theoretical analysis in Ref. [18], the linear relationship is independent of aberration magnitude and kind. The relationship remains valid in principle under strong turbulence levels. In practical experiments, however, strong turbulence results in a more dispersed and fragmented intensity distribution. This phenomenon diminishes the energy concentration on the imaging-based photoelectric detector and deteriorates the signal-to-noise ratio. Since the masked intensity signals are directly obtained from the detector information, the increased noise restricts the estimation accuracy, particularly for low-order modes with large amplitudes. Therefore, the observed performance degradation is mainly attributed to measurement noise rather than a fundamental breakdown of the linear model. Despite this degradation, the proposed method maintains overall convergence and stability up to $D/r_{0}=20$ under our experimental conditions.

Finally, the experimental system functions as a laboratory-scale demonstration for correcting quasi-static wavefront aberrations generated by the Roddier method. The measured iteration time of approximately 0.67 s is constrained by both hardware and software limitations rather than the control algorithm itself. Specifically, hardware factors, such as the finite response time of DM, the readout time of CMOS, and software overhead from data acquisition and control signal computation in MATLAB, restrict the attainable iteration rate. Potential approaches for real-time implementation include faster detectors and deformable mirrors, underlying software environment, and parallel processing using FPGA or GPU platforms. Although the experiments are carried out in a limited laboratory environment, the demonstrated robustness under large aberrations indicates promising applicability to more realistic scenarios.

## 6. Conclusions

To address the limitations of conventional wavefront sensorless adaptive optics systems in terms of convergence speed and computational efficiency, this paper proposes and implements a model-based wavefront sensorless adaptive optics experimental setup.

Experimental results under different turbulence levels demonstrate that the model-based wavefront sensorless adaptive optics system can achieve rapid and effective wavefront correction with a relatively small number of system iterations. Compared to the SPGD algorithm, the model-based method exhibits a significantly faster convergence under the identical hardware and software configuration. Under weak turbulence conditions, the time required by the SPGD algorithm is 4.77 times that of the model-based method, and this ratio increases to approximately 14.53 times under strong turbulence conditions. Moreover, as the turbulence levels increases, the advantages of the model-based method in terms of convergence speed and stability become increasingly evident, indicating enhanced robustness and broader applicability. In short, the model-based wavefront sensorless adaptive optics system shows greater potential for correcting large wavefront aberrations under strong turbulence levels, offering theoretical support and technical guidance for dynamic aberration correction and real-time control in complex atmosphere turbulence environments. The applicability of the model-based method to dynamically varying turbulence will be investigated in our next work.

## Data Availability

Data underlying the results presented in this paper are not publicly available at this time but may be obtained from the authors upon reasonable request.
